# NFL is a marker of treatment response in children with SMA treated with nusinersen

**DOI:** 10.1007/s00415-019-09389-8

**Published:** 2019-05-23

**Authors:** Bob Olsson, Lars Alberg, Nicholas C. Cullen, Eva Michael, Lisa Wahlgren, Anna-Karin Kroksmark, Kevin Rostasy, Kaj Blennow, Henrik Zetterberg, Már Tulinius

**Affiliations:** 1 Department of Psychiatry and Neurochemistry, Institute of Neuroscience and Physiology, Sahlgrenska Academy at the University of Gothenburg, V-house, Sahlgrenska University Hospital Mölndal, 431 80 Mölndal, Sweden; 2 Clinical Neurochemistry Laboratory, Sahlgrenska University Hospital, Mölndal, Sweden; 3 Queen Silvia Children’S Hospital, Sahlgrenska University Hospital, Gothenburg, Sweden; 4 Department of Neurology, Perelman School of Medicine, University of Pennsylvania, Philadelphia, PA USA; 5 Institute of Health and Care Sciences, Sahlgrenska Academy at the University of Gothenburg, Gothenburgs, Sweden; 6 Pediatric Neurology, Children’S Hospital Datteln, Witten/Herdecke University, Datteln, Germany; 7 Department of Neurodegeneration, UCL Institute of Neurology, Queen Square, London, UK; 8 UK Dementia Research Institute at UCL, London, UK; 9 Department of Pediatrics, Institute of Clinical Sciences, Sahlgrenska Academy at the University of Gothenburg, Gothenburg, Sweden

**Keywords:** SMA, Cerebrospinal fluid, Biomarkers, Neurofilament, Tau

## Abstract

**Background:**

Recently, the anti-sense oligonucleotide drug nusinersen was approved for spinal muscular atrophy (SMA) and our aim was to find a response marker for this treatment.

**Methods:**

Twelve children with SMA type 1 and two copies of the *SMN2* gene were included in a consecutive single-center study. The children were sampled for CSF at baseline and every time nusinersen was given intrathecally. The neuronal biomarkers NFL and tau and the glial biomarker GFAP were measured. Motor function was assessed using CHOP INTEND. Eleven similarly aged children, who were investigated to rule out neurological or infectious disease, were used as controls.

**Results:**

Baseline levels of NFL (4598 ± 981 vs 148 ± 39, *P* = 0.001), tau (939 ± 159 vs 404 ± 86, *P* = 0.02), and GFAP (236 ± 44 vs 108 ± 26, *P* = 0.02) were significantly higher in SMA children than controls. Motor function improved by nusinersen treatment in median 13 points corresponding to 5.4 points per month of treatment (*P* = 0.001). NFL levels typically normalized ( < 380 pg/ml) between the fourth and fifth doses [− 879.5 pg/mL/dose, 95% CI (− 1243.4, − 415.6), *P* = 0.0001], tau levels decreased [− 112.6 pg/mL/dose, 95% CI (− 206–7, − 18.6), *P* = 0.01], and minor decreases in GFAP were observed [− 16.9 pg/mL/dose, 95% CI (− 22.8, − 11.2), *P* = 0.02] by nusinersen treatment. Improvement in motor function correlated with reduced concentrations of NFL (rho = − 0.64, *P* = 0.03) and tau (rho = − 0.85, *P* = 0.0008) but not GFAP.

**Conclusions:**

Nusinersen normalized the axonal damage marker NFL and correlated with motor improvement in children with SMA. NFL may, therefore, be a novel biomarker to monitor treatment response early in the disease course.

## Introduction

Spinal muscular atrophy (SMA) is an autosomal recessive disease that causes degeneration of spinal cord motor neurons resulting in hypotonia and muscle weakness [1]. The carrier frequency of SMA is one in 50 and the incidence is one in 10,000. The most severe form of the disease is SMA type one (SMA1; approximately 50% of affected children), which is characterized by an onset before 6 months of age and a life expectancy of less than 2 years [1]. Children with SMA type two (SMA2) have a milder form of the disease, with an onset between 6 and 18 months of age, can sit without support, and a few can stand with leg braces, but none can walk independently. Difficulty in swallowing and coughing and respiratory insufficiency may occur during adolescence for individuals with SMA2. Patients with SMA type three (SMA3) have onset of disease after 18 months. They learn to walk, although some require wheelchair assistance later in life; patients with SMA type four (SMA4) suffer from milder motor impairment and are able to walk in adult years.

Patients with SMA have homozygous non-functional copies of the survival motor neuron 1 gene (*SMN1)* at locus 5q13 [2]. Due to a duplication, the gene *SMN2* exists in zero to five copies. The *SMN2* gene is identical to the *SMN1* gene except for five base pairs [2]. Of these five, the single-nucleotide base change in exon 7 causes alternative splicing and omission of exon 7 in *SMN2*, which in turn leads to a truncated form of the protein that is rapidly degraded [3]. Even so, approximately 5–10% of the *SMN2* gene is still transcribed with exon 7 intact and a viable protein as result [2].

Nusinersen is an anti-sense oligonucleotide drug which prevents omission of exon 7 and subsequent loss of motor function by targeting the pre-mRNA of *SMN2* [4, 5]. Thereby, the protein levels of SMN increases and motor neurons are saved [6]. Two phase 3 clinical trials have shown the effectiveness of nusinersen. In the first trial, infants were included [4], and in the second, children with symptoms arising after the age of 6 months were included [5]. Regardless of onset, both trials demonstrated that the groups treated with nusinersen improved in motor function, whereas the children in the sham control groups deteriorated further. However, a few children in both trials did not respond to treatment [4, 5]. Thus, it would be valuable to have a biomarker that could predict treatment response early on. Nusinersen was recently approved for treatment of SMA by the FDA and EMA.

Microtubule-associated protein tau and neurofilament light protein (NFL) are two neuron-specific structural proteins. Tau is currently used as a clinical cerebrospinal fluid (CSF) biomarker of cortical neuronal degeneration in Alzheimer’s disease [7], but increased levels are also found with other types of neuronal damage, e.g., Creutzfeldt-Jakob disease and stroke [8]. NFL, on the other hand, together with its medium and heavy counterpart makes up the intermediate axonal filaments that determine axonal caliber and in part axonal velocity and is a well-established CSF biomarker for neurodegeneration across a wide range of neurodegenerative diseases [9]. Another marker is GFAP, an intermediate filament present in astrocytes [10]. Our aim was to identify a biomarker to monitor response to nusinersen treatment in children with SMA type 1. For this, we measured CSF NFL, tau, and GFAP at baseline, and consecutively, in children with SMA at each timepoint, the drug was given.

## Methods

### Patients

Twelve children with SMA type 1 and two copies of the *SMN2* gene were consecutively recruited in this single-center study (Table 1). Motor function was measured with Children's Hospital of Philadelphia Infant Test of Neuromuscular Disorders (CHOP INTEND) [11]. CHOP INTEND is a validated 16-item motor assessment designed specifically to evaluate the motor skills of infants with SMA; higher score indicates greater motor skill. The total CHOP INTEND score ranges 0–64. Motor function was assessed at start of treatment and thereafter at intervals of between 2 to 6 months. Eleven children sampled for facial nerve palsy or to exclude meningitis or cerebellitis were used as controls. The study was approved by the Regional Ethics Committee in Gothenburg and all parents gave informed consent.

**Table 1:** Demographics of SMA children

Characteristic	
*N*	12
Sex	
Male	4
Female	8
Age at onset (months)	2.2 ± 1.4 [0.3, 4]
Age at treatment (months)	14.4 ± 24.0 [1.2, 92.4]
Baseline Motor Score	24.3 ± 8.2 [11, 38]
NFL Levels (pg/mL)	
Dose 1	4598.3 ± 3398.1 (n = 12)
Dose 2	4020.0 ± 2792.3 (n = 10)
Dose 3	3173.3 ± 2067.0 (n = 9)
Dose 4	1289.0 ± 1232.1 (n = 10)
Dose 5	331.0 ± 223.9 (n = 10)
Dose 6	161.2 ± 87.4 (n = 8)
Dose 7	106.7 ± 47.3 (n = 3)
Dose 8	215.0 ± 176.8 (n = 2)
Tau Levels (pg/mL)	
Dose 1	939.5 ± 551.1 (n = 12)
Dose 2	802.6 ± 372.4 (n = 10)
Dose 3	788.4 ± 368.7 (n = 9)
Dose 4	528.1 ± 225.4 (n = 10)
Dose 5	462.0 ± 260.9 (n = 10)
Dose 6	454.6 ± 304.5 (n = 8)
Dose 7	165.7 ± 57.5 (n = 3)
Dose 8	166.0 ± 123.1 (n = 2)
GFAP Levels (pg/mL)	
Dose 1	235.8 ± 153.4 (n = 12)
Dose 2	214.0 ± 89.1 (n = 10)
Dose 3	228.9 ± 72.0 (n = 9)
Dose 4	206.0 ± 137.4 (n = 10)
Dose 5	147.0 ± 70.7 (n = 10)
Dose 6	166.2 ± 95.5 (n = 8)
Dose 7	130.0 ± 65.6 (n = 3)
Dose 8	105.0 ± 49.5 (n = 2)

All values are mean ± SD. Numbers in brackets indicate range. The motor score was measured with CHOP INTEND

### CSF analyses

CSF was sampled at baseline, and every time, the drug was given intrathecally at the Queen Silvia Children’s Hospital, Sahlgrenska University Hospital. Five mL of CSF was collected in polypropylene tubes, centrifuged (2000×*g* for 10 min), and sent to the Clinical Neurochemistry Laboratory, Sahlgrenska University Hospital for storage at − 80 ℃ (within 2 h from sampling). Biomarker measurements were performed in clinical laboratory practice on a weekly basis using methods and procedures accredited by the Swedish Board for Accreditation and Conformity Assessment (SWEDAC). The measurements were performed by board-certified laboratory technicians who were blinded to clinical data. The first three doses were given every 2 weeks, the fourth dose 4 weeks later, and the following doses every 4 months. NFL, tau, and GFAP concentrations were measured by enzyme-linked immunosorbent assays (the NF-light kit from UmanDiagnostics, Umeå, Sweden, the INNOTEST hTau kit from Fujirebio, Gent, Belgium and an in-house ELISA for GFAP [12]).

### Statistical analysis

Patient #6 has continued treatment abroad after the first two doses. She increased to 24 points in CHOP INTEND after further dosing, but since we did not have access to the corresponding CSF samples, we have only included the baseline value in the analyses. We did not have access to CSF sample #3 for patient #9 due to the fact that this dose was given at the child’s local hospital. CSF sample #2 for patient #10 was lost. Eight patients received six doses, three patients received seven doses, and two patients received eight doses of nusinersen before data cut (Tables 1 and 2).

**Table 2:** Individuals in the study and treatment regimen

Patient	Group	Sex	Age at onset (months)	Age at baseline (months)	Number of doses received
1	SMA	M	0	0.5	5
2	SMA	M	1	2	5
3	SMA	F	1	3	6
4	SMA	F	1	3	8
5	SMA	M	1	4	1
6	SMA	F	2	18	2
7	SMA	F	2	92	7
8	SMA	F	3	7	6
9	SMA	M	3	12	8
10	SMA	M	4	14	6
11	SMA	F	4	8	6
12	SMA	F	4	9	6
1	Control	F	NA	36	NA
2	Control	F	NA	30	NA
3	Control	F	NA	4	NA
4	Control	F	NA	26	NA
5	Control	M	NA	3	NA
6	Control	M	NA	6	NA
7	Control	M	NA	18	NA
8	Control	M	NA	23	NA
9	Control	M	NA	10	NA
10	Control	M	NA	48	NA
11	Control	M	NA	96	NA

Differences in baseline values of biomarkers between children with SMA and controls were analyzed with ANCOVA with age and sex as covariates.

Change in motor function as measured by the CHOP INTEND score was first analyzed independent of biomarker concentrations using linear mixed effects modelling with varying intercepts and varying slopes. Days since first nusinersen treatment was used as the longitudinal dimension and individuals were treated as a random effect. Age at onset and age at treatment were included in separate interaction terms with the time dimension in order on to analyze their effects on both baseline and change in motor function. Spearman rank correlation coefficients were also calculated between intercepts/slopes and change in motor function.

Changes in CSF biomarker levels over time were analyzed using linear mixed effects modelling with varying intercepts and varying slopes, since we expected both baseline levels and treatment effect to differ between individuals. Sex, age at disease onset, and age at treatment were included fixed effects in the model, while individuals were treated as a random effect. Dose number was used as the longitudinal dimension for biomarker analysis, since dose timing was relatively standard across individuals and since models were adjusted for both age at onset and age at treatment. No adjustment for baseline biomarker levels was included, because CSF was not sampled before start of treatment. Mixed effects modelling was chosen due to its robustness to missing values and its ability to analyze both within- and between-subject characteristics of longitudinal data.

All analyses were performed using the R programming language, version 3.4, and the mixed effects modelling was specifically carried out using the *nlme* package, version 3.1. All tests were two-sided with a statistical significance threshold of *P* < 0.05.

## Results

One patient (#5) had severe symptoms already at onset of treatment and was converted to palliative care after only one dose given (Table 2). This patient was excluded from further analysis. The remaining 11 children were treated for a mean of 11 months, ranging from 2.5 to 19 months. Patient #7 was 92 weeks of age at start of treatment. The onset of disease was at 2 months of age. She was never able to sit independently and fulfilled all criteria for SMA type 1 including having 2 SMN2 copies. The patient needed intensive pulmonary care including non-invasive ventilation by nasal mask but never needed tracheostomy. She was often hospitalized, but survived due to intensive treatment. There was no significant difference in age (*P* = 0.24) or sex distribution (*P* = 0.44) between the SMA and the control groups. Demographics are shown in Table 1.

**Fig. 1:**
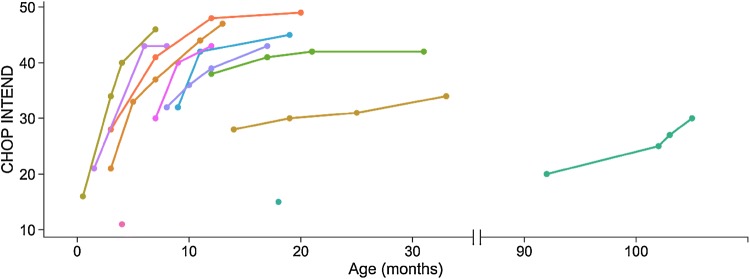
Change in motor function over time in children with SMA treated with nusinersen. Motor function improved in all patients who received more than one dose of nusinersen. The CHOP INTEND score increased 13 points in median (range 3–30 points) and 5.4 points per month of treatment on average (*P* < 0.0001). Patient #6 (only 1 CHOP INTEND value with baseline 15 points in the figure) has continued treatment abroad and increased to 24 points in CHOP INTEND after further dosing, but since we did not have access to the corresponding CSF samples, we have only included the baseline value in the figure and in calculations

Motor function improved for all patients who received more than one dose of nusinersen (patient #6 continued treatment abroad after dose two). The CHOP INTEND score increased 13 points in median (range 3–30 points), with an estimated improvement of 5.4 points per month according to mixed effects model analysis (*P* < 0.0001, Fig. 1). In addition, change in CHOP INTEND score was inversely associated with age at disease onset (*B* = − 1.53, *P* = 0.01), while age at treatment had no effect on change in motor function (*B* = − 0.56, *P* = 0.35). Age at disease onset had no relationship with baseline CHOP INTEND score (*B* = -0.92, *P* = 0.13), while age at treatment had a significant inverse relationship (*B* = − 0.92, *P* = 0.03).

CSF NFL levels in the SMA children in our study were clearly increased at baseline compared with historical control data derived from children with benign medical history, including transient headache, diffuse pain, suspected infections, or habitual change in walking pattern, where brain imaging or CSF inflammatory and infectious markers were normal [13]. Baseline levels of NFL (4598 ± 981 vs 148 ± 39, *P* = 0.001), tau (939 ± 159 vs 404 ± 86, *P* = 0.02), and GFAP (236 ± 44 vs 108 ± 26, *P* = 0.02) were also significantly higher in the SMA children than the controls in our study.

**Fig. 2:**
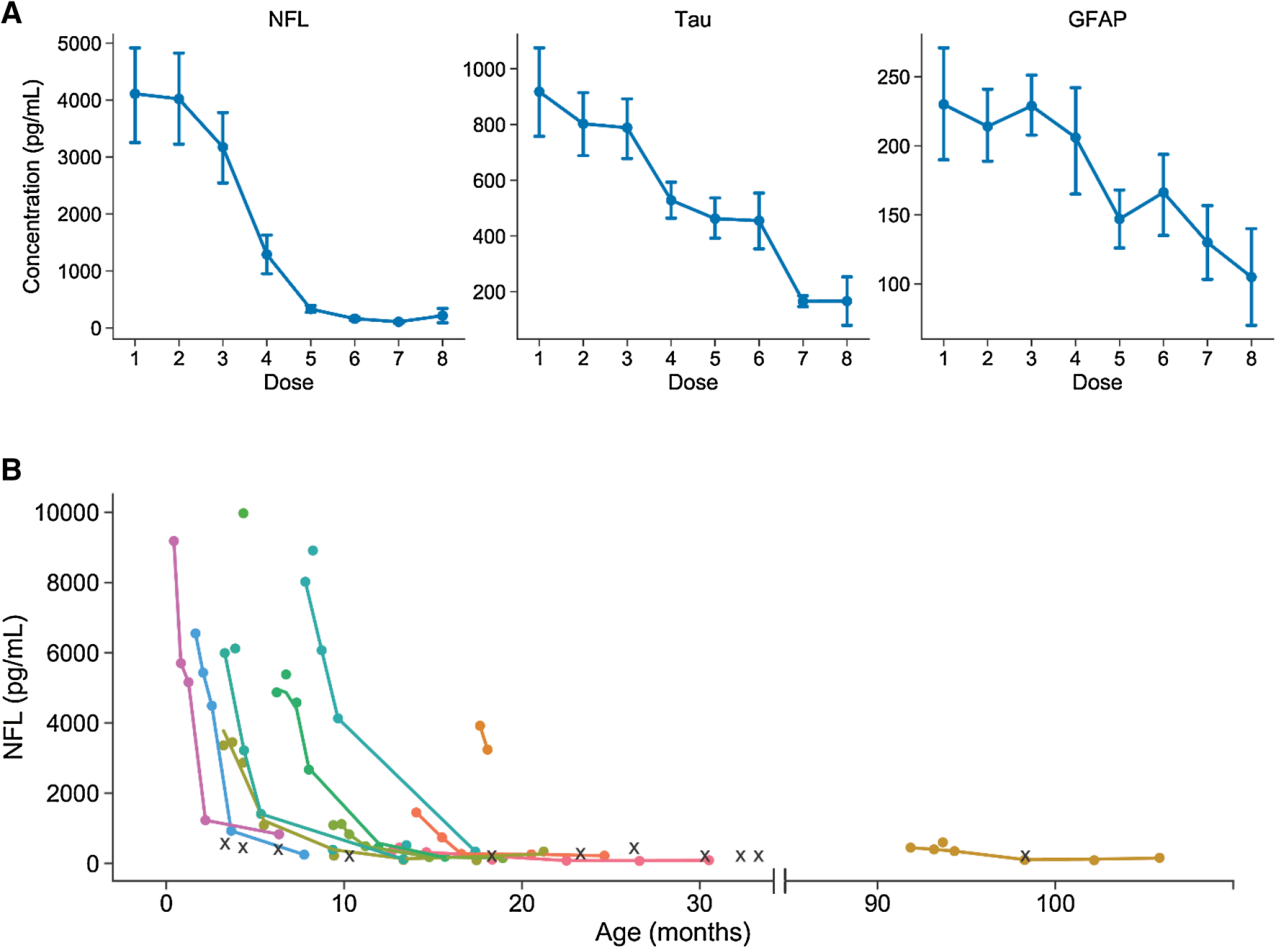
Changes in CSF NFL, tau, and GFAP concentrations in children with SMA by nusinersen treatment. **a** Changes in CSF NFL, tau, and GFAP concentrations by nusinersen treatment. NFL concentrations decreased significantly over time by nusinersen treatment [mean change = − 879.5 pg/mL per dose, 95% CI (− 1343.4, − 415.6), *P* = 0.0001] and normalized ( < 380 pg/ml) between the fourth and fifth treatments. Tau concentrations also decreased significantly over time [mean change = − 112.6 pg/mL per dose, 95% CI (− 206.7, − 18.6), *P* = 0.01] and minor but significant decreases were observed for GFAP [mean change = − 16.9 pg/mL per dose, 95% CI (− 22.8, − 11.2), *P* = 0.02]. **b** Changes in CSF NFL concentrations by age and nusinersen treatment. One patient (#5) had severe symptoms after receiving only one dose and was converted to palliative care before he died. This patient was excluded from further analysis. Each X denotes one of the 11 controls

**Fig. 3:**
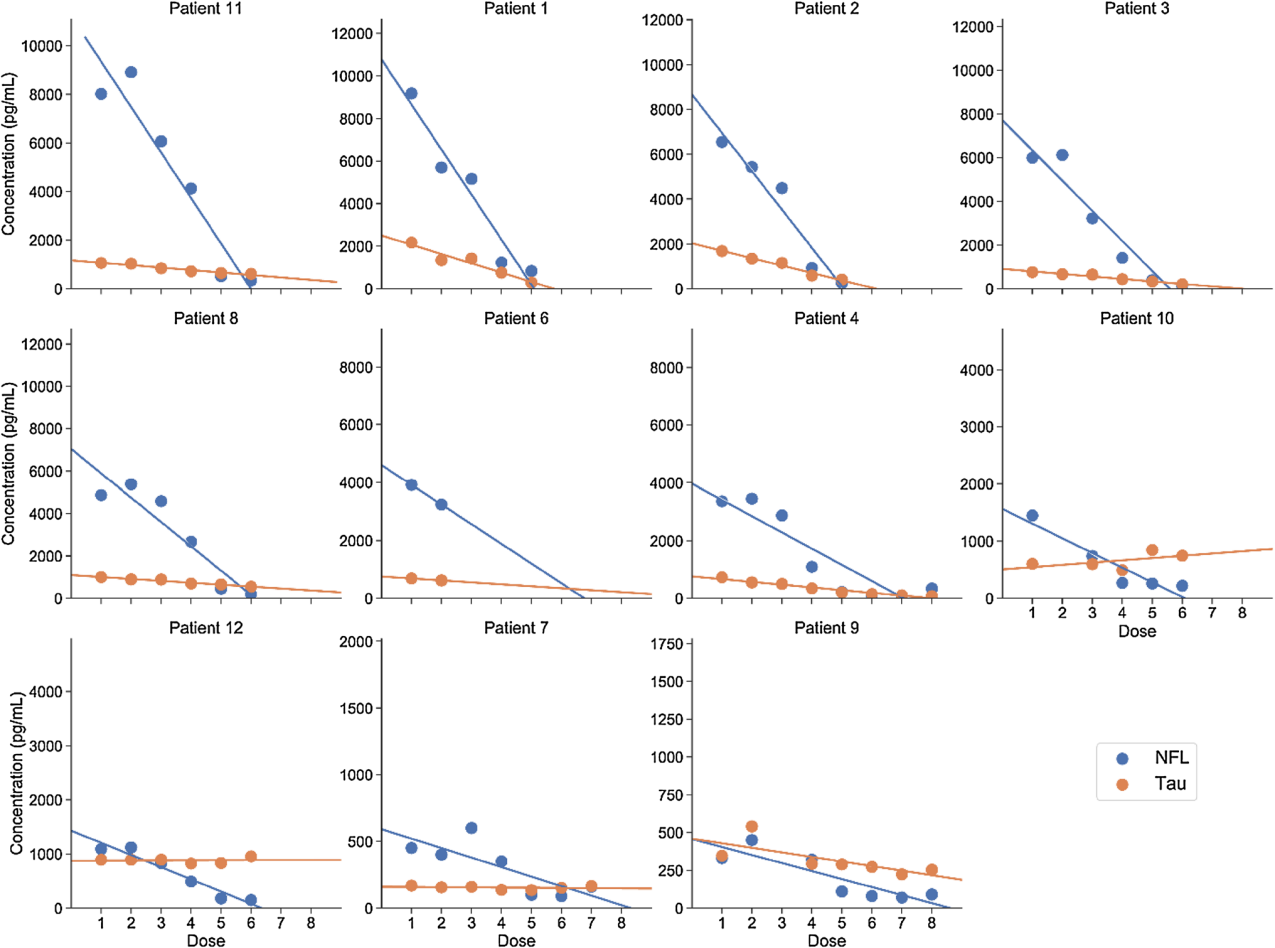
Individual changes in NFL and tau levels. One patient (#5) had severe symptoms after receiving only one dose and was converted to palliative care before he died. This patient was excluded from further analysis

NFL levels decreased over time by nusinersen treatment [mean change = − 879.5 pg/mL per dose, 95% CI (− 1343.4, − 415.6), *P* = 0.0001] and typically normalized ( < 380 pg/ml which is the cutoff in clinical routine for children [14]) between the fourth and fifth treatments (Fig. 2). Furthermore, earlier treatment initiation was associated with a larger decrease in NFL (rho = 0.67; *P* = 0.02) according to correlation analysis, with a nearly significant association according to mixed effects modelling (*B* = 154.8, *P* = 0.09). Tau levels also decreased over time [mean change = − 112.6 pg/mL per dose, 95% CI (− 206.7, − 18.6), *P* = 0.01, Fig. 2] with larger decreases in those with earlier disease onset (rho = 0.68, *P* = 0.02) and treatment initiation according to correlation analysis (rho = 0.88, *P* = 0.0003) and mixed effects modelling (*B* = 73.02, *P* = 0.002). Individual changes in NFL and tau are shown in Fig. 3. Minor decreases were observed for GFAP [mean change = − 16.9 pg/mL per dose, 95% CI (− 22.8, − 11.2), *P* = 0.02; Fig. 2] but no association with either age at onset or age at treatment. Moreover, change in motor function correlated with change in NFL (rho = − 0.64, *P* = 0.03), tau (rho = − 0.85, *P* = 0.0008) but not GFAP.

## Discussion

CSF levels of NFL normalized and correlated with motor improvement in children with the most severe form of SMA treated with nusinersen.

In ALS, motor neurons in the anterior horn of the spinal cord are destroyed leading to progressive loss of motor function and death [15]. Since motor neurons have the largest axons in the body and NFL is a subunit of neurofilaments, which is the main intermediate filament of axons, it is not surprising that CSF levels of NFL are increased in ALS as NFL is released from the injured and dying axons [16]. In some ways, SMA mirrors ALS as motor neurons also are lost in SMA. Nusinersen has been shown to ameliorate motor neuron decline in a mouse model of SMA [17]. Since NFL is an axonal marker [9] and the initial high levels of NFL that normalized by nusinersen treatment in combination with improved motor function suggest that the direct target of the disease, i.e., motor neurons, may be salvaged in children with SMA. However, future studies are needed to confirm this. A previous study of NFL in children with opsoclonus–myoclonus syndrome, non-inflammatory neurological disorders, and other inflammatory neurological disorders showed that CSF levels of NFL do not correlate with age [18]. The lack of association between age and NFL was also observed in a study by Shahim et al. [13]. Furthermore, NFL decreased in all children who received more than one dose of nusinersen regardless of age in our study. In addition, we found significantly higher levels in children with SMA1 than in control children having an age span covering the SMA1 children, demonstrating that the decrease in NFL is caused by nusinersen treatment and not due to changes in CSF biomarker levels during normal development.

It is also apparent that the greatest benefit of the drug was found in children who received treatment earliest in their disease course, as the decrease in NFL was largest in those children. This suggests that treatment should commence as early as possible, since the longer treatment is delayed the worse effect on neurons. Thus, NFL may be used to identify children in need of rapid treatment and those requiring dose escalation. Since we observed such a rapid decline and normalization in CSF levels of NFL after only a few treatments, NFL may be used as an early response marker guiding physicians whether to continue treatment or not. This may also be important for parents, since they will know early on whether their child will benefit from the treatment or continue to deteriorate.

As mentioned above, the main consequence of SMA is loss of motor neurons, the axons of which are rich in NFL. Tau, on the other hand, is generally considered a cortical neuronal marker. Baseline levels of tau were significantly higher in children with SMA than in controls, indicating that children with SMA may also suffer from cortical neuronal damage. Furthermore, tau levels decreased by nusinersen treatment, indicating that the cortical neuronal damage is alleviated by nusinersen treatment. In addition, baseline levels of GFAP were also increased in children with SMA compared with controls, indicating that astrogliosis is increased in SMA which could be ameliorated by nusinersen, since the levels of GFAP were reduced by treatment.

All children in our study who received more than one dose of nusinersen improved in motor function and this improvement correlated with decreased CSF levels of NFL and tau. As we did not have any children on placebo or sham, we refer to the clinical trials of nusinersen which show that the natural course of the disease is progressive loss of motor function [4, 5]. Thus, nusinersen treatment was the reason behind the improvement in motor function also in our study.

Since both NFL and tau correlated with motor improvement in children with SMA, one could argue that both could work as treatment markers in SMA. However, since the magnitude of the decrease was so much larger for NFL than for tau and also that more children normalized, i.e., had levels below those set for clinical routine, in their NFL levels than for tau, NFL is in our view a better treatment marker than tau in SMA. In adults, NFL levels have been shown to be elevated in most forms of neurodegenerative diseases and also to correlate with clinical progression in Alzheimer’s disease, frontotemporal dementia, and Parkinson’s disease [19]. NFL concentrations also correlate with measures of cognition and brain atrophy in Huntington’s disease [20], indicating that it might be a promising marker for upcoming treatment regimes in diseases beside SMA.

The limitations of this study include a small study population which could have influenced some of the results such as the non-significant result for change in motor function in relation to age at start of treatment. Still, we have included all available children with SMA type 1 in Sweden, since the drug was approved in late 2016. A strength of this study is that this was a single-center study and all children were treated by the same physician.

In conclusion, CSF levels of NFL normalized and correlated with motor improvement in children with SMA type 1 treated with nusinersen. NFL may, therefore, be a novel biomarker to monitor treatment response to nusinersen early in the disease course. Furthermore, our data suggest that treatment should commence at the earliest timepoint to save as much of motor neurons as possible.

## References

[1] Lunn MR, Wang CH (2008). Spinal muscular atrophy. Lancet.

[2] Lefebvre S, Burglen L, Reboullet S, Clermont O, Burlet P, Viollet L, Benichou B, Cruaud C, Millasseau P, Zeviani M (1995). Identification and characterization of a spinal muscular atrophy-determining gene. Cell.

[3] Lorson CL, Androphy EJ (2000). An exonic enhancer is required for inclusion of an essential exon in the SMA-determining gene SMN. Hum Mol Genet.

[4] Finkel  RS, Mercuri E, Darras BT, Connolly AM, Kuntz NL, Kirschner  J, Chiriboga CA, Saito  K, Servais L, Tizzano E, Topaloglu  H, FamilyName>Tulinius M, Montes J, Glanzman AM, Bishop  K,  Zhong  ZJ, Gheuens  s, Bennett  CF, Schneider  E,  Farwell  W, De Vivo  DC, Group ES (2017). Nusinersen versus sham control in infantile-onset spinal muscular atrophy. N Engl J Med.

[5] Mercuri  E, Darras BT, Chiriboga CA, Day JW,  Campbell C, Connolly AM, Iannaccone ST,  Kirschner  J, Kuntz NL, Saito K, Shieh PB, Tulinius M, Mazzone  ES,  Montes J, Bishop KM, Yang  Q, Foster  R, Gheuens S, Bennett CF, Farwell W,  Schneider E, De Vivo DC, Finkel RS, Group CS (2018). Nusinersen versus sham control in later-onset spinal muscular atrophy. N Engl J Med.

[6] Hua Y, Sahashi K, Rigo F, Hung G, Horev G, Bennett CF, Krainer AR (2011). Peripheral SMN restoration is essential for long-term rescue of a severe spinal muscular atrophy mouse model. Nature.

[7] Olsson B, Lautner R, Andreasson U, Ohrfelt A, Portelius E, Bjerke M, Holtta M, Rosen C, Olsson C, Strobel G, Wu E, Dakin K, Petzold M, Blennow K, Zetterberg H (2016). CSF and blood biomarkers for the diagnosis of Alzheimer's disease: a systematic review and meta-analysis. Lancet Neurol.

[8] Blennow K, Hampel H, Weiner M, Zetterberg H (2010). Cerebrospinal fluid and plasma biomarkers in Alzheimer disease. Nat Rev Neurol.

[9] Zetterberg H (2016). Neurofilament light: a dynamic cross-disease fluid biomarker for neurodegeneration. Neuron.

[10] Bignami A, Eng LF, Dahl D, Uyeda CT (1972). Localization of the glial fibrillary acidic protein in astrocytes by immunofluorescence. Brain Res.

[11] Glanzman AM, Mazzone E, Main M, Pelliccioni M, Wood J, Swoboda KJ, Scott C, Pane M, Messina S, Bertini E, Mercuri E, Finkel RS (2010). The children's hospital of Philadelphia infant test of Neuromuscular disorders (CHOP INTEND): test development and reliability. Neuromuscul Disord.

[12] Rosengren LE, Wikkelso C, Hagberg L (1994). A sensitive ELISA for glial fibrillary acidic protein: application in CSF of adults. J Neurosci Methods.

[13] Shahim P, Darin N, Andreasson U, Blennow K, Jennions E, Lundgren J, Mansson JE, Naess K, Tornhage CJ, Zetterberg H, Mattsson N (2013). Cerebrospinal fluid brain injury biomarkers in children: a multicenter study. Pediatr Neurol.

[14] Yilmaz A, Blennow K, Hagberg L, Nilsson S, Price RW, Schouten J, Spudich S, Underwood J, Zetterberg H, Gisslen M (2017). Neurofilament light chain protein as a marker of neuronal injury: review of its use in HIV-1 infection and reference values for HIV-negative controls. Expert Rev Mol Diagn.

[15] Kiernan MC, Vucic S, Cheah BC, Turner MR, Eisen A, Hardiman O, Burrell JR, Zoing MC (2011). Amyotrophic lateral sclerosis. Lancet.

[16] Rosengren LE, Karlsson JE, Karlsson JO, Persson LI, Wikkelso C (1996). Patients with amyotrophic lateral sclerosis and other neurodegenerative diseases have increased levels of neurofilament protein in CSF. J Neurochem.

[17] Passini MA, Bu J, Richards AM, Kinnecom C, Sardi SP, Stanek LM, Hua Y, Rigo F, Matson J, Hung G, Kaye EM, Shihabuddin LS, Krainer AR, Bennett CF, Cheng SH (2011). Antisense oligonucleotides delivered to the mouse CNS ameliorate symptoms of severe spinal muscular atrophy. Sci Transl Med.

[18] Pranzatelli MR, Tate ED, McGee NR, Verhulst SJ (2014). CSF neurofilament light chain is elevated in OMS (decreasing with immunotherapy) and other pediatric neuroinflammatory disorders. J Neuroimmunol.

[19] Olsson B, Portelius E, Cullen NC, Sandelius A, Zetterberg H, Andreasson U, Hoglund K, Irwin DJ, Grossman M, Weintraub D, Chen-Plotkin A, Wolk D, McCluskey L, Elman L, Shaw LM, Toledo JB, McBride J, Hernandez-Con P, Lee VM, Trojanowski JQ, Blennow K (2018) Association of cerebrospinal fluid neurofilament light protein levels with cognition in patients with dementia, motor neuron disease, and movement disorders. JAMA Neurol 76:318–325. 10.1001/jamaneurol.2018.3746. [Epub ahead of print] 10.1001/jamaneurol.2018.3746 PMC6440232 30508027

[20] Byrne LM, Rodrigues FB, Blennow K, Durr A, Leavitt BR, Roos RAC, Scahill RI, Tabrizi SJ, Zetterberg H, Langbehn D, Wild EJ (2017). Neurofilament light protein in blood as a potential biomarker of neurodegeneration in Huntington's disease: a retrospective cohort analysis. Lancet Neurol.

